# Evaluation of the Environmental Fate of a Semivolatile Transformation Product of Ibuprofen Based on a Simple Two-Media Fate Model

**DOI:** 10.1021/acs.est.2c04867

**Published:** 2022-10-14

**Authors:** Cecilia Arsene, Iustinian G. Bejan, Claudiu Roman, Romeo I. Olariu, Marco Minella, Monica Passananti, Luca Carena, Davide Vione

**Affiliations:** † Department of Chemistry, Faculty of Chemistry, “Alexandru Ioan Cuza” University of Iasi, 11 Carol I, 700506Iasi, Romania; ‡ Integrated Centre of Environmental Science Studies in the North Eastern Region (CERNESIM), “Alexandru Ioan Cuza” University of Iasi, 11 Carol I, 700506Iasi, Romania; § Integrated Centre of Environmental Science Studies in the North Eastern Region (RECENT AIR), “Alexandru Ioan Cuza” University of Iasi, 11 Carol I, 700506Iasi, Romania; ∥ Dipartimento di Chimica, Università degli Studi di Torino, Via Pietro Giuria 5, 10125Torino, Italy; ⊥ Institute for Atmospheric and Earth System Research/Physics, Faculty of Science, University of Helsinki, FI-00014Helsinki, Finland

**Keywords:** hydroxyl radicals, 4-isobutylacetophenone, rate coefficient, water−air interface, aqueous system modeling, chemodynamics, environmental modeling

## Abstract

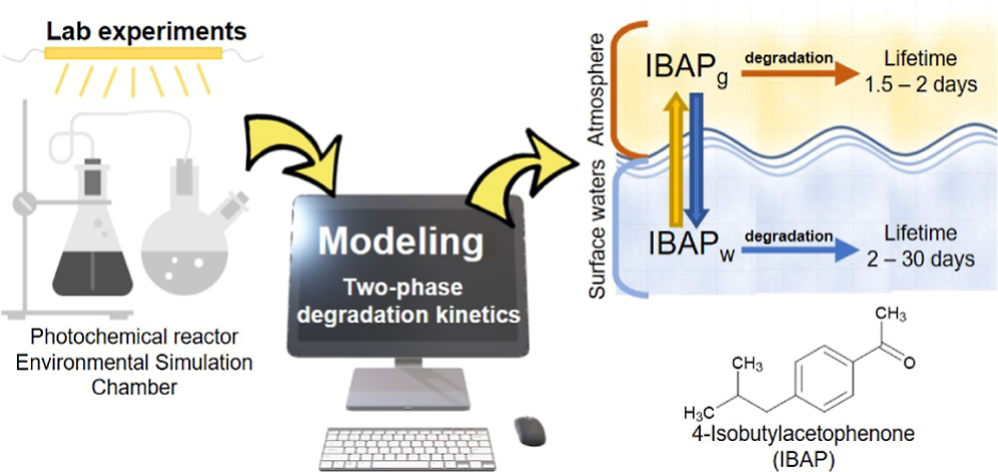

Partitioning between surface waters and the atmosphere is an important process, influencing the fate and transport of semi-volatile contaminants. In this work, a simple methodology that combines experimental data and modeling was used to investigate the degradation of a semi-volatile pollutant in a two-phase system (surface water + atmosphere). 4-Isobutylacetophenone (IBAP) was chosen as a model contaminant; IBAP is a toxic transformation product of the non-steroidal, anti-inflammatory drug ibuprofen. Here, we show that the atmospheric behavior of IBAP would mainly be characterized by reaction with ^•^OH radicals, while degradation initiated by ^•^NO_3_ or direct photolysis would be negligible. The present study underlines that the gas-phase reactivity of IBAP with ^•^OH is faster, compared to the likely kinetics of volatilization from aqueous systems. Therefore, it might prove very difficult to detect gas-phase IBAP. Nevertheless, up to 60% of IBAP occurring in a deep and dissolved organic carbon-rich water body might be eliminated *via* volatilization and subsequent reaction with gas-phase ^•^OH. The present study suggests that the gas-phase chemistry of semi-volatile organic compounds which, like IBAP, initially occur in natural water bodies in contact with the atmosphere is potentially very important in some environmental conditions.

## Abstract 2

This paper addresses the environmental fate of semi-volatile and toxic 4-isobutylacetophenone in a coupled water−atmosphere system, showing that volatilization followed by gas-phase degradation cannot be neglected.

## Introduction

The problems connected with the pollution of environmental compartments by human activities would be much worse than currently experienced, if self-cleaning processes did not take place. The self-cleaning ability of the atmosphere can be largely explained by physico-chemical processes, including the chemical degradation of pollutants by oxidative reactive species [*e.g.*, hydroxyl radicals (^•^OH) during the day and nitrate radicals (^•^NO_3_) during the night], as well as dry and wet pollutant deposition.^1,2^ Moreover, some atmospheric pollutants (especially those having C=C double bonds) are also scavenged by reaction with ozone,^3^ while others undergo important direct photolysis (degradation upon sunlight absorption by the pollutant itself).^1^

The mentioned oxidative reactive species (*e.g.*, ^•^OH, ^•^NO_3_, and O_3_) are responsible for initiating the degradation of air pollutants and are mainly generated through atmospheric photochemical processes.^4−6^ Atmospheric ^•^OH is mainly produced by HONO photolysis in the early morning^7^ and later on by sunlight irradiation of HCHO.^8,9^ Moreover, depending on atmospheric conditions, sunlight UVB irradiation of ozone,^10^ reaction between ozone and alkenes, and reaction between nitric oxide (^•^NO) and a photogenerated hydroperoxide radical (HO_2_^•^) might also be important sources of tropospheric ^•^OH.^11,12^^•^OH can also be formed indoors, mostly upon HONO photolysis.^13,14^

O_3_ is generated photochemically as well following the reaction between ^•^OH and alkenes and ^•^NO_2_ photolysis. ^•^NO_3_ is formed by the reaction between O_3_ and ^•^NO_2_, which reach their highest concentration values during the day but still occur in the night. The ^•^NO_3_ formation rate is actually the highest during the day, but it is offset by very fast ^•^NO_3_ photolysis (with the exception of tree canopies, which provide sufficient shading and are among the few environments where ^•^NO_3_ can be detected in daytime^15^).

Pollutants can be biodegraded in surface waters, but biorecalcitrant contaminants preferentially undergo photodegradation by direct photolysis or by reaction with the so-called photochemically produced reactive intermediates (PPRIs). The main PPRIs consist in ^•^OH again, plus the carbonate radical (CO_3_^•–^), the triplet states of chromophoric dissolved organic matter (^3^CDOM*), and singlet oxygen (^1^O_2_). PPRIs are produced by photosensitizers, which are (mostly) naturally occurring compounds that generate PPRIs upon sunlight absorption.^16,17^ Main surface-water photosensitizers are nitrate and nitrite (direct sources of ^•^OH and indirect CO_3_^•–^ sources upon oxidation of HCO_3_^–^/CO_3_^2–^ by photogenerated ^•^OH), as well as CDOM. The latter produces ^•^OH, ^3^CDOM*, and also ^1^O_2_ upon reaction between ^3^CDOM* and dissolved O_2_. Indirectly, irradiated CDOM yields CO_3_^•–^, again *via*^•^OH and also *via* oxidation of CO_3_^2–^ by ^3^CDOM*.^17−20^ Note that iron is often listed among the photosensitizers too.^21^ However, given the poor occurrence of dissolved Fe(III) hydroxo species at the typical pH values of most environmental waters (with the major exceptions of strongly acidified lakes and acidic mine drainage) and because of low photoreactivity of colloidal Fe(III) (hydr)oxides, iron mostly contributes to the chromophoric nature of CDOM in the form of organic complexes.^22^ PPRIs are very efficiently scavenged/quenched in natural surface waters (^•^OH by DOM, HCO_3_^–^, and CO_3_^2–^; CO_3_^•–^ by DOM; ^3^CDOM* by O_2_; and ^1^O_2_ by collision with water).^17,23,24^ As a result of the latter processes, photoreactions may be slower in sunlit surface waters than in the atmosphere.

Surface waters and the atmosphere are often studied separately. However, they have high potential to cooperate in transport and degradative removal of semi-volatile pollutants, which partition between both phases. In the case of semi-volatile compounds, it is important to consider their joint fate in both the hydrosphere and the atmosphere to predict potential global distillation effects and/or degradation rates in the environment. Still, the combined atmospheric and surface-water fates of semi-volatile contaminants are rarely taken into account together or compared.

In this work, we chose 4-isobutylacetophenone (hereinafter, IBAP) as a model contaminant, which is a toxic transformation product of the very popular non-steroidal, anti-inflammatory drug ibuprofen (IBP). IBP has been detected in natural waters at concentration levels ranging from ng L^–1^ to μg L^–1^.^25−28^ IBAP has been detected in river water at ng L^–1^ levels,^29^ and photochemical modeling suggests that its concentration could amount to about 15% of that of IBP.^30^

Transformation of IBP into IBAP accounts for the adverse health effects of expired IBP formulations, and IBAP is also produced by IBP photochemistry in sunlit surface waters following direct photolysis and ^3^CDOM* reaction of the parent compound (and, to a much lesser extent, IBP degradation by ^•^OH).^31^ IBAP is semi-volatile, and it could also undergo partitioning from surface waters to the atmosphere. To the best of our knowledge, IBAP reactivity in an atmospheric context is totally unknown, differently from IBAP photochemical fate in sunlit waters. Therefore, this contribution has the following goals: (i) to measure IBAP reactivity with the main gas-phase atmospheric oxidants and (ii) to provide, through modeling, an overall assessment of IBAP fate in a two-phase environmental compartment (surface water + atmosphere).

## Materials and Methods

### Chemicals Used in the Gas-Phase Kinetic Study

Purchased chemicals: IBAP 97% (Alfa Aesar); dimethyl ether (DME) >99.9% (cylinder, Sigma-Aldrich); cyclohexane (CyHex) >99.5% (Sigma-Aldrich); *p*-benzoquinone (*p*-Bq) >98% (Sigma-Aldrich); 2,3-dimethyl-2-butene >98% (Aldrich); ^•^NO >99.5% (cylinder, Linde); synthetic air 99.999% (cylinder, Messer); and oxygen 99.999% (cylinder, Messer). Ozone was generated by passing a flow of oxygen over a Hg VUV lamp in a separate flow tube connected to the reaction vessel. CH_3_ONO was produced from methanol >99% (Sigma-Aldrich) and KNO_2_ (Sigma-Aldrich) in an acidic solution as described by Taylor *et al.*,^32^ and it was stored as a gas in an opaque glass gas cylinder. ^•^NO_3_ radicals were obtained *in situ* from the reaction of O_3_ with ^•^NO_2_ >99% (cylinder, Linde).

The starting concentrations (in molecules cm^–3^) of the compounds transferred into the reaction vessel were as follows: (3.49–6.75) × 10^13^ for IBAP; (4.87–8.12) × 10^13^ for DME; (2.92–4.37) × 10^13^ for CyHex; (8.72–10.02) × 10^13^ for *p*-Bq; (9.74–16.24) × 10^13^ for CH_3_ONO; 16.24 × 10^13^ for ^•^NO; and (2.46–4.92) × 10^13^ for O_3_. For the free-NO_*x*_ condition kinetic study, addition of 1 μL of 2,3-dimethyl-2-butene (tetramethylethylene, TME) by direct syringe injection into the smog chamber (*vide infra*) was preferred to ensure considerable ^•^OH radical production in the gas-phase system.

### Gas-Phase Reactivity of IBAP

The gas-phase kinetic study of IBAP with ^•^OH radicals, both with and without NO_*x*_ (the latter termed as the NO_*x*_-free condition), was performed in a simulated atmosphere, at 298 ± 3 K and 1 bar pressure of synthetic air, using the 760 L ESC-Q-UAIC (environmental simulation chamber—made of quartz—from the “Alexandru Ioan Cuza” University of Iasi, Romania) chamber facilities (for a more detailed description, see ref (33)). In the NO_*x*_-containing system, irradiation was carried out with lamps having an emission maximum at 365 nm to achieve *in situ* generation of ^•^OH radicals from CH_3_ONO. Photolysis of IBAP was investigated at both 365 and 254 nm. A Bruker Vertex 80 spectrometer (MCT-N_2_-cooled detector), connected to a white-type mirror system (producing an optical path of 492 ± 1 m inside the reactor), was used as the main analytical tool to monitor the sink of reactants during kinetic experiments. The IR spectra were recorded every minute, with a spectral resolution of 1 cm^–1^, as an average of 60 scans per output spectrum.

Gas-phase reaction rate constants of IBAP with ^•^OH radicals [*k*_g_(IBAP + ^•^OH)] were measured, relative to three different reference compounds: DME, with *k*_g_(DME + ^•^OH) = 2.83 × 10^–12^ cm^3^ molecule^–1^ s^–1^;^34^ cyclohexane (CyHex), with *k*_g_(CyHex + ^•^OH) = 6.38 × 10^–12^ cm^3^ molecule^–1^ s^–1^;^35^ and *p*-Bq, with *k*_g_(*p*-Bq + ^•^OH) = 4.60 × 10^–12^ cm^3^ molecule^–1^ s^–1^.^36^ During kinetic investigations, gas-phase processes inside the reaction vessel were as follows
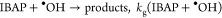
1
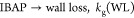
2
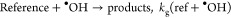
3where WL = wall loss. By evaluating the loss of the reference compounds and IBAP through processes 1–3, during the time interval *t*–*t*_0_, one gets eq 4 that describes the overall kinetics of the reaction system. Upon linearization of eq 4, one obtains the ratio *k*_g_(IBAP + ^•^OH)/*k*_g_(ref + ^•^OH) and, because *k*_g_(ref + ^•^OH) is known, one also gets the second-order rate constant for the reaction between IBAP and ^•^OH, *k*_g_(IBAP + ^•^OH).
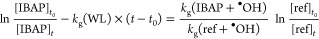
4

Preliminary tests performed in the ESC-Q-UAIC chamber showed that IBAP exhibited wall loss, with a first-order rate constant *k*_g_(WL) = (3.1 ± 0.4) × 10^–4^ s^–1^ (uncertainties here represent ±2σ). Over an irradiation time of 15 min, photolysis at 365 nm was not observed for IBAP or the reference compounds. At 254 nm, significant photolysis was observed for IBAP in ESC-Q-UAIC, with an estimated first-order rate constant of (7.5 ± 0.4) × 10^–4^ s^–1^. The photolysis rate constant was obtained from the IBAP decay, after correction for the wall-loss process as shown in Figure S1 of the Supporting Information. Because of important IBAP photolysis at 254 nm, 254 nm H_2_O_2_ photolysis could not be used as an *in situ*^•^OH radical source. Therefore, ozonolysis of TME in the dark was a good alternative to generate ^•^OH radicals in the reactor under NO_*x*_-free conditions.

Test experiments were also performed in the ESC-Q-UAIC smog chamber to preliminarily check for the gas-phase behavior of IBAP in the presence of O_3_ and ^•^NO_3_. None of the performed tests indicated contributions from other processes, apart from wall loss, which excludes significant reactivity between IBAP and ^•^NO_3_ or O_3_.

The reaction of ozone with alkenes produces ^•^OH radicals.^37^ TME is known as an important ^•^OH precursor, with a yield (*Y*) close to unity.^12,38^ By considering the amount of ozone occurring in the reactor, the ^•^OH radical concentration can be estimated as follows^39^
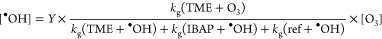
5

Concentrations as high as 4 × 10^8^ radicals cm^–3^ can be estimated for the ^•^OH radicals, taking into account the recommended values of the reaction rate constants of TME + ^•^OH, *k*_g_(TME + ^•^OH) = 1.1 × 10^–10^ cm^3^ molecule^–1^ s^–1^; TME + O_3_, *k*_g_(TME + O_3_) = 1.1 × 10^–15^ cm^3^ molecule^–1^ s^–1^;^34^ and the ozone concentration range of (2.46–4.92) × 10^13^ molecule cm^–3^.

### Modeling of IBAP Photodegradation in Surface Waters and in Two-Phase (Surface Waters + Atmosphere) Systems

**Scheme 1:**
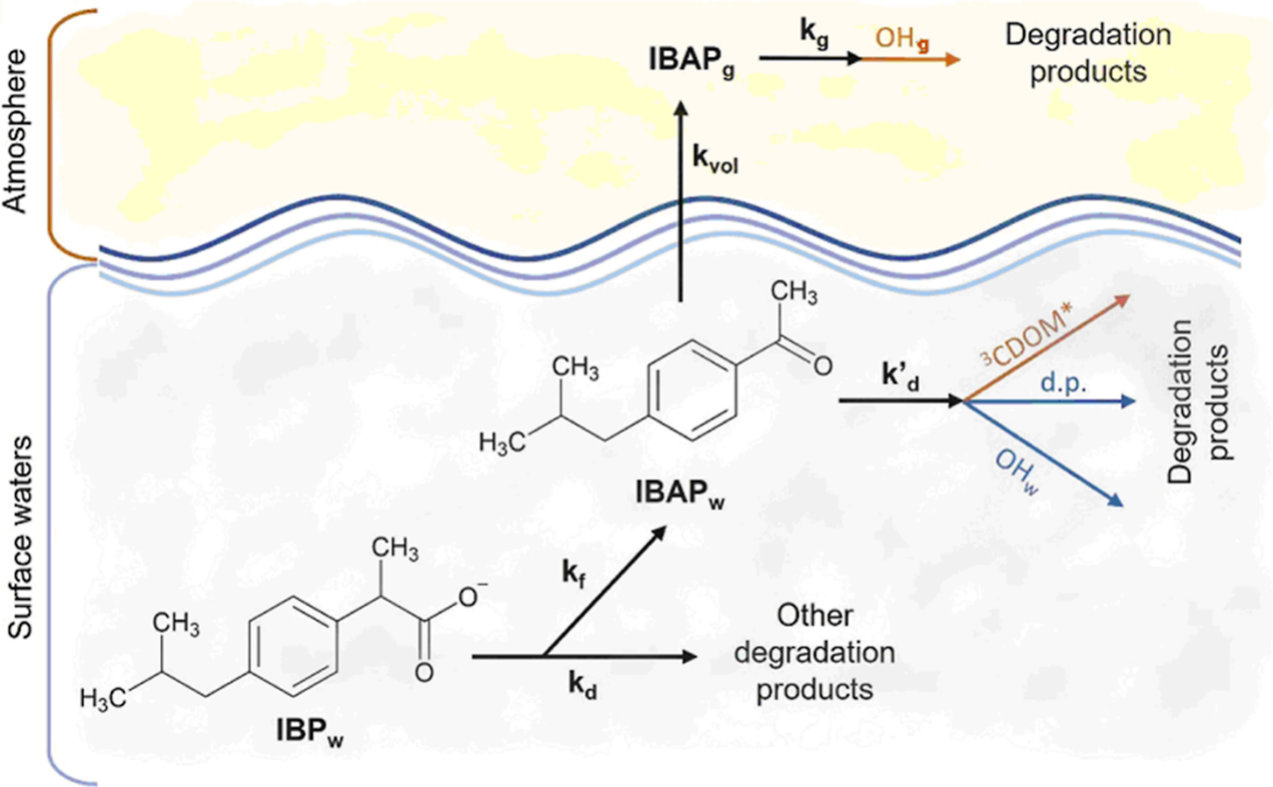
Overall Schematic of IBP Phototransformation into IBAP, Followed by IBAP Degradation in the Aqueous Phase and by IBAP Partitioning to the Gas Phase with Subsequent Degradation by ^•^OH_(g)_ The (pseudo)first-order rate constants are shown above each relevant arrow and are discussed in the text. In particular, *k*_g_ was obtained from the data of gas-phase IBAP reactivity with ^•^OH as *k*_g_ = *k*_g_(IBAP + ^•^OH) [^•^OH_(g)_] (reaction 9, *vide supra*); *k*_vol_ was estimated with EPISuite, while *k*_d_, *k*_f_, and *k*_d_^′^ were assessed by means of APEX modeling. Degradation pathways of IBAP in blue and orange are favored under “fast kinetics” and “slow kinetics” scenarios, respectively.

The overall two-phase reaction pathways involving IBP and IBAP are depicted in Scheme 1. IBP initially occurs in surface water (IBP is typically emitted by urban wastewater treatment plants due to incomplete degradation) as the carboxylate form, thus its air–water partitioning can be neglected. Conversely, photochemistry plays an important role in the attenuation of IBP in surface waters.^45^ A significant fraction of photodegraded IBP is accounted for by IBAP,^40,41^ which is initially formed in aqueous solution (IBAP_w_). IBAP can then undergo either water-phase photodegradation or partitioning to the gas phase, where it is mostly degraded by ^•^OH_(g)_ as mentioned before.

From the reactions given in Scheme 1, one derives the following expressions for the time trends of IBP_w_, IBAP_w_, and IBAP_g_, where [IBP_w_]_o_ is the initial concentration of IBP in aqueous solution^42^
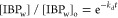
6
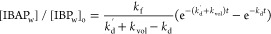
7
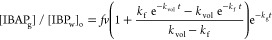
8where *k*_d_ is the first-order degradation rate constant of IBP in water, *k*_d_^′^ that of IBAP, *k*_f_ the formation rate constant of IBAP from IBP, and *k*_vol_ the volatilization rate constant of IBAP. The quantity *f* = *k*_f_*k*_d_^–1^ is the fraction of IBP_w_ that is transformed into IBAP_w_, and  is the fraction of IBAP that undergoes volatilization to the gas phase.

The values of *k*_f_, *k*_d_, and *k*_d_^′^ were modeled with the APEX software.^43^ APEX can model the direct and indirect photochemistry of pollutants in well-mixed surface waters, such as the whole water column of lakes during overturn, the lake epilimnion during summer stratification, and even shallow systems like flooded rice fields.^44^ APEX modeling requires knowledge of key environmental features of the water body [contents of dissolved organic carbon (DOC), nitrate, nitrite, carbonate, and bicarbonate, as well as water depth] and of photoreactivity parameters of the pollutant(s) under consideration. The latter include direct photolysis quantum yields, second-order rate constants for the reactions with the different PPRIs (^•^OH, ^1^O_2_, and ^3^CDOM*), and, for IBAP as an intermediate, formation yields from IBP in the different reaction pathways.

These parameters have been measured experimentally in previous work, for each photoreaction pathway,^30,45^ and are summarized in Table 1. The photochemical lifetimes computed by APEX refer to mid-July and mid-latitude irradiation conditions.

**Table 1:** Photoreactivity Parameters, Relevant to the Photodegradation of IBP and IBAP in Surface Freshwaters and to the Phototransformation of IBP into IBAP^45^ a,b

	IBP	IBAP
Φ, mol Einstein^–1^	0.33	5.0 × 10^–2^
*k*_^•^OH_, M^–1^ s^–1^	1.0 × 10^10^	2.0 × 10^10^
*k*_^1^O_2__, M^–1^ s^–1^	6.0 × 10^4^	2.3 × 10^6^
*k*_^3^CDOM*_, M^–1^ s^–1^	1.5 × 10^9^	3.2 × 10^9^
η_IBP→IBAP_^d.p.^, unitless	0.25	
η_IBP→IBAP_^^•^OH^, unitless	0.023	
η_IBP→IBAP_^^1^O_2_^, unitless	negligible	
η_IBP→IBAP_^^3^CDOM*^, unitless	0.31	

a They were used as input data for the APEX software.

b Φ: direct photolysis quantum yield; *k*: second-order reaction rate constant; η: formation yield of IBAP from IBP.

The pseudo-first-order reaction rate constant between IBAP and gas-phase ^•^OH (*k*_g_) was derived from experimental reactivity data (see previous section) as the second-order reaction rate constant between IBAP_(g)_ and ^•^OH_(g)_ [*k*_g_(IBAP + ^•^OH)] times the typical, 24 h averaged values of [^•^OH_(g)_]. In particular,
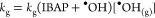
9

The volatilization rate constant of IBAP from aqueous environments (*k*_vol_) was determined with EPISuite^46^ using a quantitative structure–activity relationship (SAR) approach. The volatilization model followed the method described by Thomas (1990),^47^ assuming relatively calm wind conditions (0.5 m s^–1^ velocity).

Text S1 (Supporting Information) suggests that this approach works better than one based on gas–water partitioning equilibrium (Henry’s law), which holds only if volatilization is much faster than degradation in water. In the case of IBAP, the two processes have comparable kinetics (*vide supra*), and the present approach based on first-order kinetics, without partitioning equilibrium, is to be preferred.

## Results and Discussion

### Gas-Phase Reactivity of IBAP upon Reaction with ^•^OH

**Figure 1:**
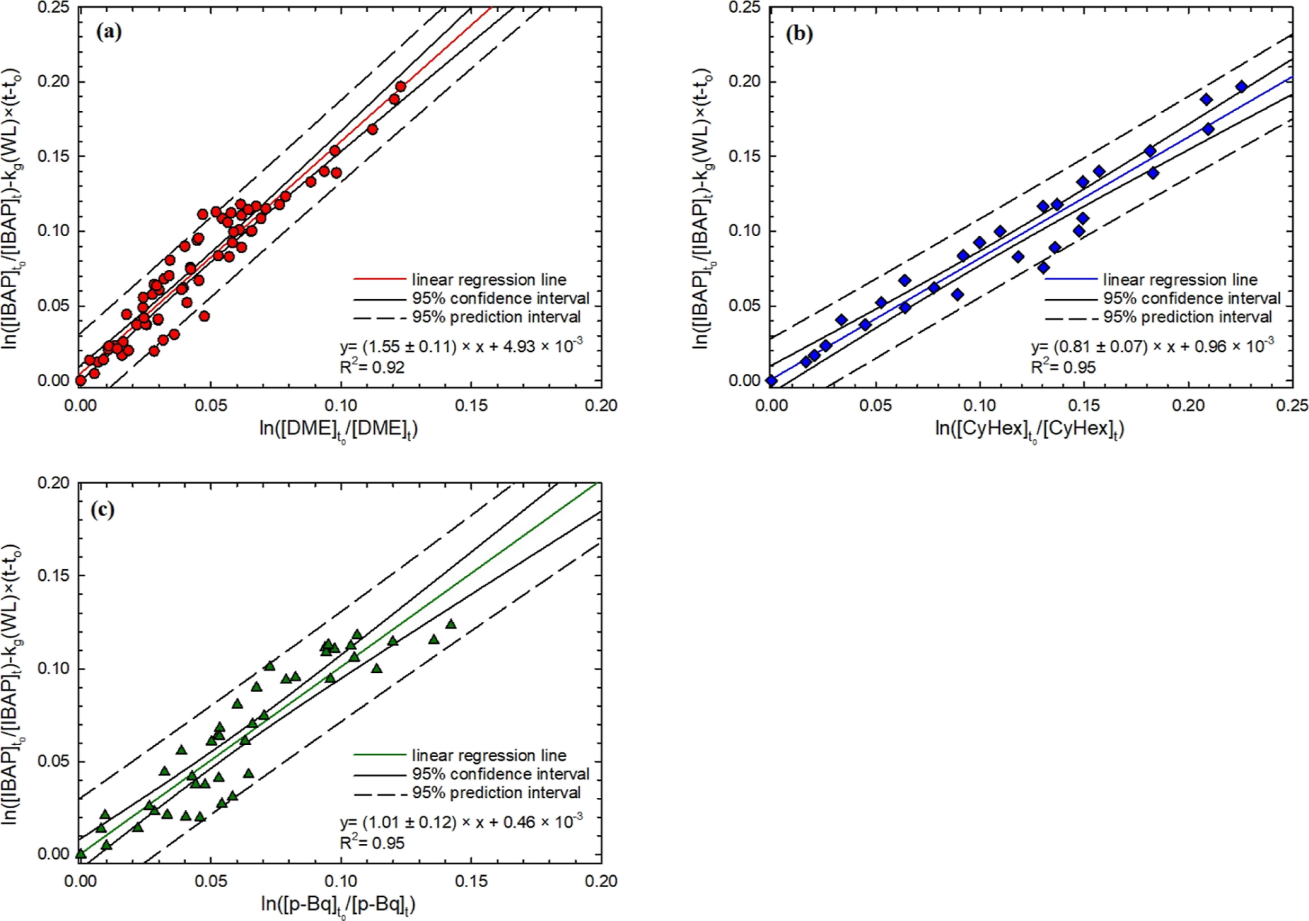
Relative kinetic plots of the IBAP gas-phase ^•^OH-initiated reaction, measured in the presence of NO_*x*_, and relative to (a) (red shaded ○) DME, (b) (blue shaded ◇) CyHex, and (c) (green shaded △) *p*-Bq according to eq 4.

**Figure 2:**
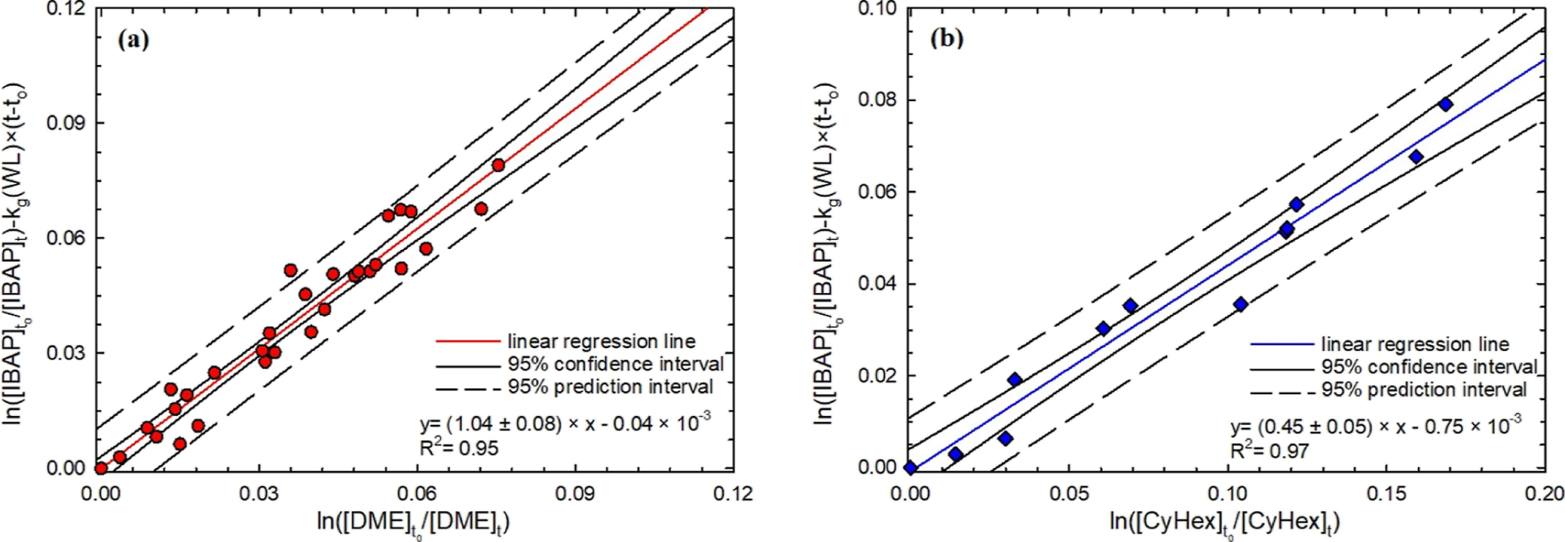
Relative kinetic plots of the IBAP gas-phase ^•^OH-initiated reaction, measured in the absence of NO_*x*_, relative to (a) (red shaded ○) DME and (b) (blue shaded ◇) CyHex according to eq 4.

Figures 1 and 2 present the results of the relative kinetics experimental data, obtained from the study of the reaction between IBAP and gas-phase ^•^OH. The reaction rate constants of IBAP with ^•^OH in the presence of NO_*x*_ were measured using three different reference compounds (*i.e.*, dimethyl ether, DME; cyclohexane, CyHex; and *para*-benzoquinone, *p*-BQ), while in the absence of NO_*x*_, only two reference compounds (DME and CyHex) were used. The slopes of the experimental lines provide the ratios of IBAP reaction rate constant versus reference rate constant (eq 4). In both Figures 1 and 2, data linearity is very good, despite the difficulties risen up by the subtraction procedure (to account for wall loss) and the relatively low conversion rate during experiments. The total conversion of IBAP was about 40%, with half of it caused by ^•^OH reactions and half caused by wall-loss processes. Control experiments were performed to check for the reliability of the reference compounds. Figure S2 (Supporting Information) shows the relative kinetic plots obtained for the ^•^OH reactions with CyHex and *p*-BQ, with DME as the reference compound. This control test showed that the ratios between the rate constants for the reactions between CyHex and ^•^OH and *p*-Bq and ^•^OH, measured relative to DME, are 1.95 ± 0.23 and 1.73 ± 0.12, respectively, in good agreement (within 15%, which is very acceptable) with literature data (2.25 ± 0.32 and 1.63 ± 0.23, respectively).^34−36^

**Figure 3:**
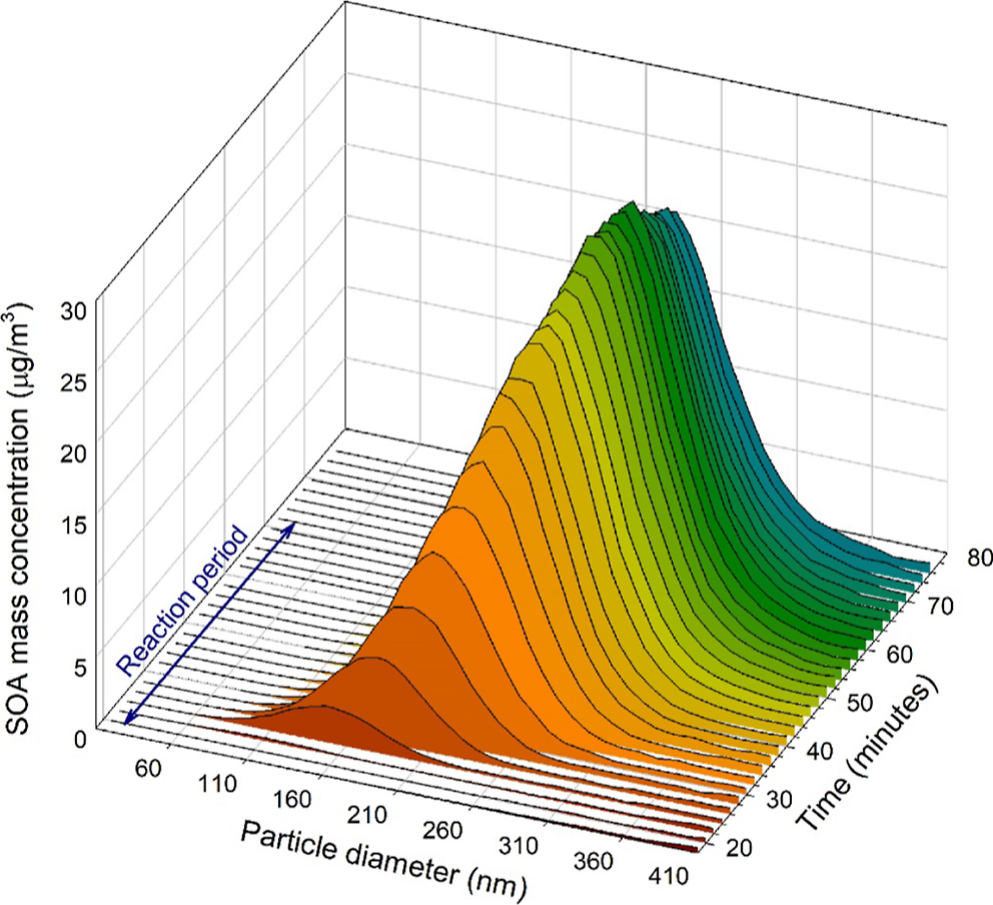
Evolution of the mass concentration of SOA and SOA particle diameter distribution, as a function of the reaction time, during IBAP photo-oxidation under ^•^OH/NO_*x*_ conditions, in the ESC-Q-UAIC chamber.

Additional results, from the ^•^OH-initiated degradation of IBAP in the gas phase, are presented in Supporting Information. Figure S3 (Supporting Information) shows a typical time evolution profile of the IBAP concentration and of the concentration (in terms of mass and particle number) of the secondary organic aerosol (SOA) formed under ^•^OH/NO_*x*_ photo-oxidation. Figure 3 shows a typical profile of the mass concentration of the organic aerosols as a function of particle diameter and reaction time.

Figure S4 (Supporting Information) underlines the secondary nature of the detected particles. From these experiments, we concluded that 5% of the total consumed carbon from gas-phase IBAP could be found in the form of SOA. These results show that IBAP has potential as a possible SOA precursor, upon reaction with ^•^OH in the gas phase. However, this datum should be considered with caution, and other experiments are needed to completely elucidate the connection between IBAP degradation in the gas phase and SOA formation.

The list of the kinetic values determined within the present study for the IBAP reaction with ^•^OH radicals, both in the presence and absence of NO_*x*_, is provided in Table 2. Uncertainties associated to the ratios among rate constants, *k*_g_(IBAP)/*k*_g_(ref), represent ±σ, obtained from linear regression analysis. The errors for the individual *k*_g_(IBAP) rate constants include an additional uncertainty of 10%, propagated from the recommended *k*_g_(ref + ^•^OH) values.

**Table 2:** Experimental Kinetic Results, Obtained from the Investigation of the IBAP Reaction with ^•^OH Radicals in the Gas Phase, Both in the Presence and Absence of NO_*x*_, along with SAR Estimates of the Same Rate Constants, as Well as Mean IBAP Lifetimes in the Atmosphere (Estimated on the Basis of Experimental Rate Constant Data)

conditions	reference	*k*_g_(IBAP)/*k*_g_(ref)	*k*_g_(IBAP) (10^–12^ cm^3^ molecule^–1^ s^–1^)	*k*_g_(IBAP)_AVG_(10^–12^ cm^3^ molecule^–1^ s^–1^)	*k*_g_(IBAP)_SAR_(10^–12^ cm^3^ molecule^–1^ s^–1^)	τ_^•^OH_(IBAP) (days)
NO_*x*_-environment	DME	1.55 ± 0.11	4.40 ± 0.53	4.67 ± 0.36	8.60^48^	2.2^46,48,49^
	CyHex	0.81 ± 0.07	5.17 ± 0.70			
	*p*-Bq	1.01 ± 0.12	4.63 ± 0.71		8.39^46^	
NO_*x*_-free environment	DME	1.04 ± 0.08	2.95 ± 0.37	2.91 ± 0.28	4.97^49^	3.5^30^
	CyHex	0.45 ± 0.05	2.87 ± 0.42			

In the presence of NO_*x*_, the rate constant value for the reaction between IBAP and ^•^OH is (4.67 ± 0.36) × 10^–12^ cm^3^ molecule^–1^ s^–1^, if estimated as the weighted average of *k*_g_(IBAP + ^•^OH) (the corresponding uncertainty was calculated accordingly). The lower rate coefficient value of (2.91 ± 0.28) × 10^–12^ cm^3^ molecule^–1^ s^–1^ was estimated for the reaction of IBAP with ^•^OH in the absence of NO_*x*_. No literature datum is available for comparison unfortunately. As a reference for comparison, Table 2 reports some SAR estimated values of *k*_g_(IBAP + ^•^OH), together with the mean lifetime of IBAP in the atmosphere, due to ^•^OH reactions. The SAR estimates were calculated by using the approach exploited in EPISuite–AOPWIN software, as proposed in Calvert *et al.*,^48^ as well as in Jenkin *et al.*(49) The EPISuite software was developed by US-EPA according to the study by Kwok and Atkinson.^50^ The reasonable agreements, especially with the newest SAR approach developed by Jenkin *et al.*,^49^ increase the confidence regarding the experimentally determined *k*_g_(IBAP + ^•^OH) (additional details are presented below).

The atmospheric lifetime of IBAP, τ_OH_(IBAP), was determined using eq 10, by assuming a 24 h average atmospheric [^•^OH] = 1.13 × 10^6^ radicals cm^–3^.^51^ From this ^•^OH concentration value, one gets that 2–3 days would be required to decrease the atmospheric concentration of IBAP by an exponential factor (*i.e.*, by ∼2.7 times). Due to quite fast reaction kinetics in the gas phase, IBAP can be considered as a local pollutant, the availability of which is spatially bounded to its main sources.
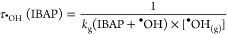
10

A difference of about 37% was observed between the IBAP rate constants with and without NO_*x*_. Although the rate constants are similar, the value measured in the absence of NO_*x*_ is significantly lower. On the one hand, in the presence of NO_*x*_, the experimental conditions inside the smog chamber are such that IBAP reactivity might be enhanced by reaction with peroxy radicals, formed from the hydrogen abstraction channel from alkylic carbon, followed by reaction with O_2_.^52^ On the other hand, concerning the NO_*x*_-free conditions, only very few studies report on the use of the reaction of TME with ozone as the ^•^OH radical source.^53^ TME has high reactivity toward ^•^OH, which introduces a limitation to the use of this ^•^OH source in reactions with rate constants higher than 10^–12^ cm^3^ molecule^–1^ s^–1^. For further discussion, we consider the rate coefficient obtained with the highest NO_*x*_ concentrations, which could represent a lower limit for the gas-phase persistence of IBAP.

To our best knowledge, this is the first study that evaluates the gas-phase rate constant of IBAP with ^•^OH. In the literature, gas-phase rate constants can be found for a similar compound, 4-methylacetophenone (MAP).^54^ MAP has a rate constant of about (4.50 ± 0.43) × 10^–12^ cm^3^ molecule^–1^ s^–1^,^54^ which almost overlaps with the rate constants determined here for IBAP. MAP has been studied with a relative rate method, in the presence of NO_*x*_, using the 365 nm photolysis of a CH_3_ONO/NO mixture as the ^•^OH radical source. This similar rate constant can be related to the structural similarity of MAP and IBAP, which both have a deactivated aromatic ring due to the presence of the keto group.

As a comparison with the IBAP–MAP pair, we could also compare the reactivity of toluene, with *k*_g_(toluene + ^•^OH) = (5.6 ± 1.46) × 10^–12^ cm^3^ molecule^–1^ s^–1^, and cumene, with *k*_g_(cumene + ^•^OH) = (6.3 ± 1.89) × 10^–12^ cm^3^ molecule^–1^ s^–1^.^34^ These compounds have slightly higher rate constants compared with IBAP and MAP, probably because their respective para positions are available for possible ^•^OH attack. Furthermore, hydrogen abstraction from different aliphatic substituents may be the cause of further reactivity differences between IBAP and MAP.

The SAR-estimated values mentioned above were obtained by using three different approaches, that is, Calvert *et al.*,^48^ EPISuite–AOPWIN,^34^ and Jenkin *et al.*(49) Two of them (EPISuite^34^ and Calvert *et al.*(48)) have been derived from the algorithm by Kwok and Atkinson.^50^ In these two cases, one gets an overestimate of IBAP rate constant (see Table 2), most likely due to the absence of electrophilic substituent constants (σ^+^) orienting in a meta position, which should have a positive value and decrease Σ(σ^+^) in the expression of the addition channel: log(*k*) = −11.71 – 1.34 × Σ(σ^+^).^48^ Moreover, these two estimated SAR values predict that both ^•^OH-addition and H-atom abstraction channels have similar contributions (∼50–50%). The third estimate was obtained by applying the model developed by Jenkin *et al.*(49) Using this SAR algorithm, a value of about 4.97 × 10^–12^ cm^3^ molecule^–1^ s^–1^ was generated, which agrees very well with our experimental finding, within experimental uncertainty. This third SAR model is based on updated factors, and it considers all ring positions as possible attack sites for the ^•^OH radicals. In this model, only 22% of the overall gas-phase ^•^OH radical reactions with IBAP is considered to proceed *via*^•^OH addition to the aromatic ring. This percentage seems to be an appropriate estimate because of deactivation of the aromatic ring by the keto group.

### IBAP Formation and Photodegradation: Gas Phase *vs* Aqueous Solution

The volatilization rate constant of IBAP from an aqueous environment to the gas phase was assessed as *k*_vol_ = 0.052 day^–1^ (EPISuite; US EPA, 2021^46^). By using the experimentally measured *k*_g_(IBAP + ^•^OH) = 4.7 × 10^–12^ cm^3^ molecules^–1^ s^–1^ and assuming a 24 h averaged [^•^OH_(g)_] = 1.13 × 10^6^ molecules cm^–3^,^30,46^ we got *k*_g_ = 5.3 × 10^–6^ s^–1^ = 0.46 day^–1^. The photoreaction parameters for natural waters were obtained by photochemical modeling with the APEX software, which were (see Scheme 1) as follows: (i) the overall photodegradation rate constant of IBP (*k*_d_); (ii) the formation rate constant of IBAP from IBP (*k*_f_), as well as (iii) the overall photodegradation rate constant of IBAP (*k*_d_^′^). APEX has been able to correctly predict the attenuation kinetics of IBP in natural waters.^45^

With APEX, we computed the reaction rate constants *k*_f_, *k*_d_, and *k*_d_^′^ as a function of environmental parameters such as, most notably, water depth, DOC, and the concentration values of nitrate and nitrite.

We simulated different scenarios and, in particular, considered two extreme cases characterized by

(i) shallow waters (*d* = 1 m) with high nitrate (10^–4^ M) and nitrite (10^–6^ M) (“fast kinetics” scenario), for which low-DOC conditions were additionally considered (1 mg_C_ L^–1^);
(ii) deep waters (*d* = 10 m) with low nitrate (10^–6^ M) and nitrite (10^–8^ M) (“slow kinetics” scenario), plus high-DOC conditions (10 mg_C_ L^–1^).

**Figure 4:**
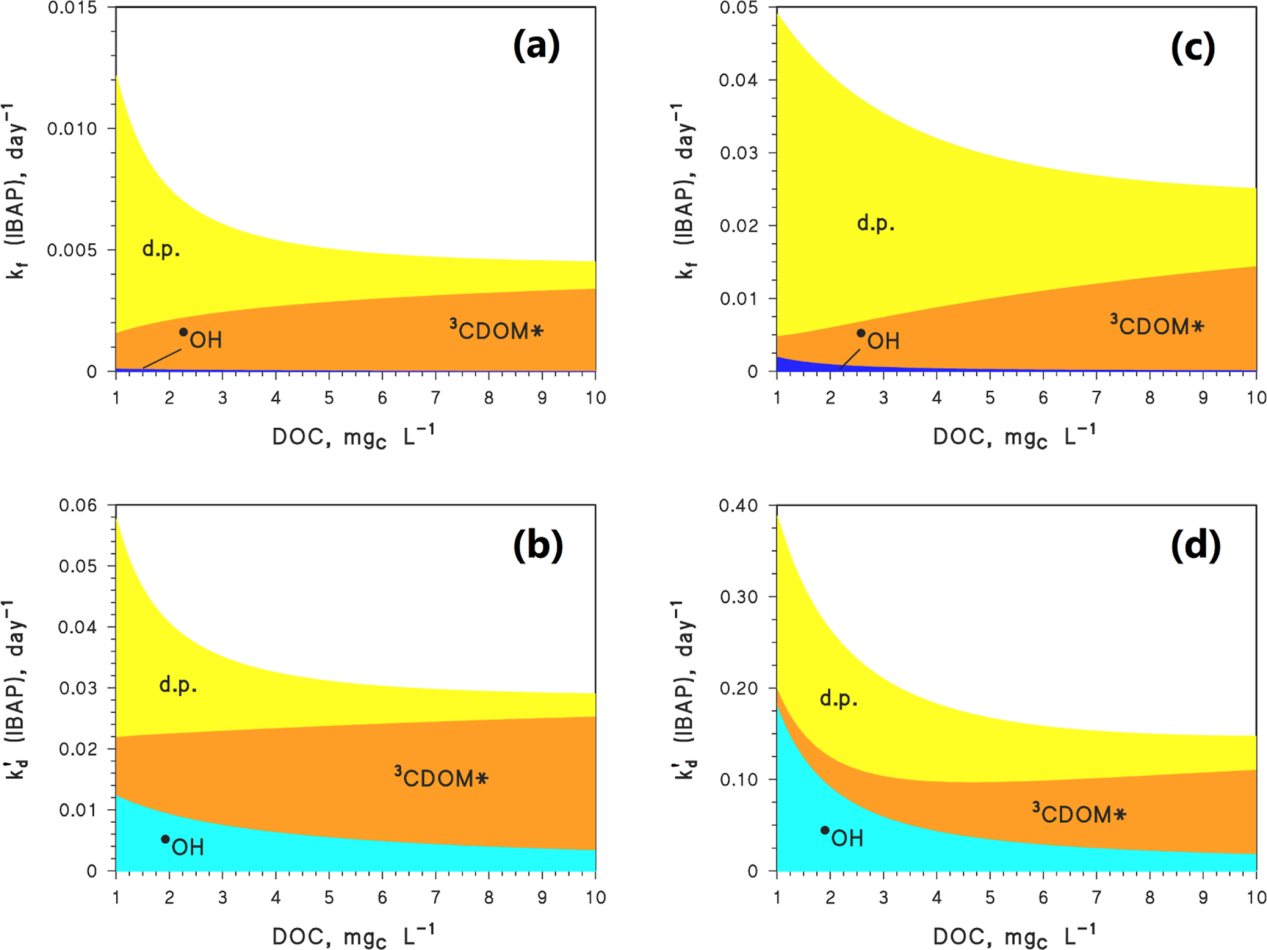
Modeled IBAP photogeneration kinetics from IBP [*k*_f_, top line, (a,c)] and IBAP photodegradation by ^•^OH, ^3^CDOM*, and the direct photolysis (d.p.) [*k*_d_^′^, bottom line, (b,d)] as a function of water DOC. The left part of the figure reports the case of slow kinetics [(a,b): deep water, low nitrate and nitrite as photochemical ^•^OH sources]; the right part reports the case of fast kinetics [(c,d): shallow water, high nitrate and nitrite, *i.e.*, high [^•^OH]]. Photochemical modeling was carried out with APEX, and the day unit refers to clear-sky 15 July at a 45°N latitude. The color code represents the importance of each photochemical pathway (^•^OH, ^3^CDOM*, direct photolysis) involved in IBAP formation, as well as degradation.

In addition to these extreme cases (Figure 4), there are, of course, intermediate conditions that we simulated as well. The relevant results are shown in the Supporting Information (Figure S5). Moreover, Figure S6 reports IBAP yield from IBP [*y*(IBP → IBAP)]. Depending on conditions, *y*(IBP → IBAP) = *k*_f_(*k*_d_)^−1^ = 0.18–0.26.

In the “fast kinetics” scenario (Figure 4c,d), fast IBAP formation is offset by fast degradation. The opposite happens in the “slow kinetics” scenario (Figure 4a,b). Photoreactions are fast in shallow waters that, differently from deep environments, are thoroughly illuminated by sunlight.

Moreover, high DOC (and high CDOM as a consequence) enhances ^•^OH scavenging and induces quenching of the direct photolysis of IBP, IBAP, nitrate, and nitrite (the latter two as ^•^OH sources), which all compete with CDOM for sunlight irradiance.

IBP and IBAP are mainly degraded by direct photolysis (d.p.) and by reactions with ^•^OH and ^3^CDOM*. Furthermore, direct photolysis and ^3^CDOM* are also involved in the formation of IBAP from IBP. Although ^•^OH is important in both IBP and IBAP degradation, it plays a minor role in IBAP formation due to the low value of  (Table 1). Therefore, high [^•^OH_(w)_] is generally detrimental to the occurrence of IBAP: in such conditions, IBP degrades fast but with relatively low IBAP production, and IBAP is degraded fast as well. Relatively high [^•^OH_(w)_] can, for instance, be attained in the presence of high-concentration values of nitrate and nitrite (hereinafter, NO_*x*_^–^), which are both photochemical ^•^OH sources.^55,56^ At high DOC, where [^•^OH_(w)_] is generally low, the ^3^CDOM* process dominates both IBAP formation and degradation (see Figure 4). Finally, the importance of the direct photolysis of either IBP or IBAP decreases as the DOC gets higher.

Overall, in the different conditions, we found that *k*_f_ would vary from 0.005 day^–1^ (*d* = 10 m, DOC = 10 mg_C_ L^–1^, low NO_*x*_^–^: 10^–6^ M NO_3_^–^ and 10^–8^ M NO_2_^–^) to 0.05 day^–1^ (*d* = 1 m, DOC = 1 mg_C_ L^–1^, high NO_*x*_^–^: 10^–4^ M NO_3_^–^ and 10^–6^ M NO_2_^–^). Under the same conditions, *k*_d_^′^ would vary from 0.03 to 0.4 day^–1^, respectively, and *k*_d_ from 0.02 to 0.3 day^–1^.

**Figure 5:**
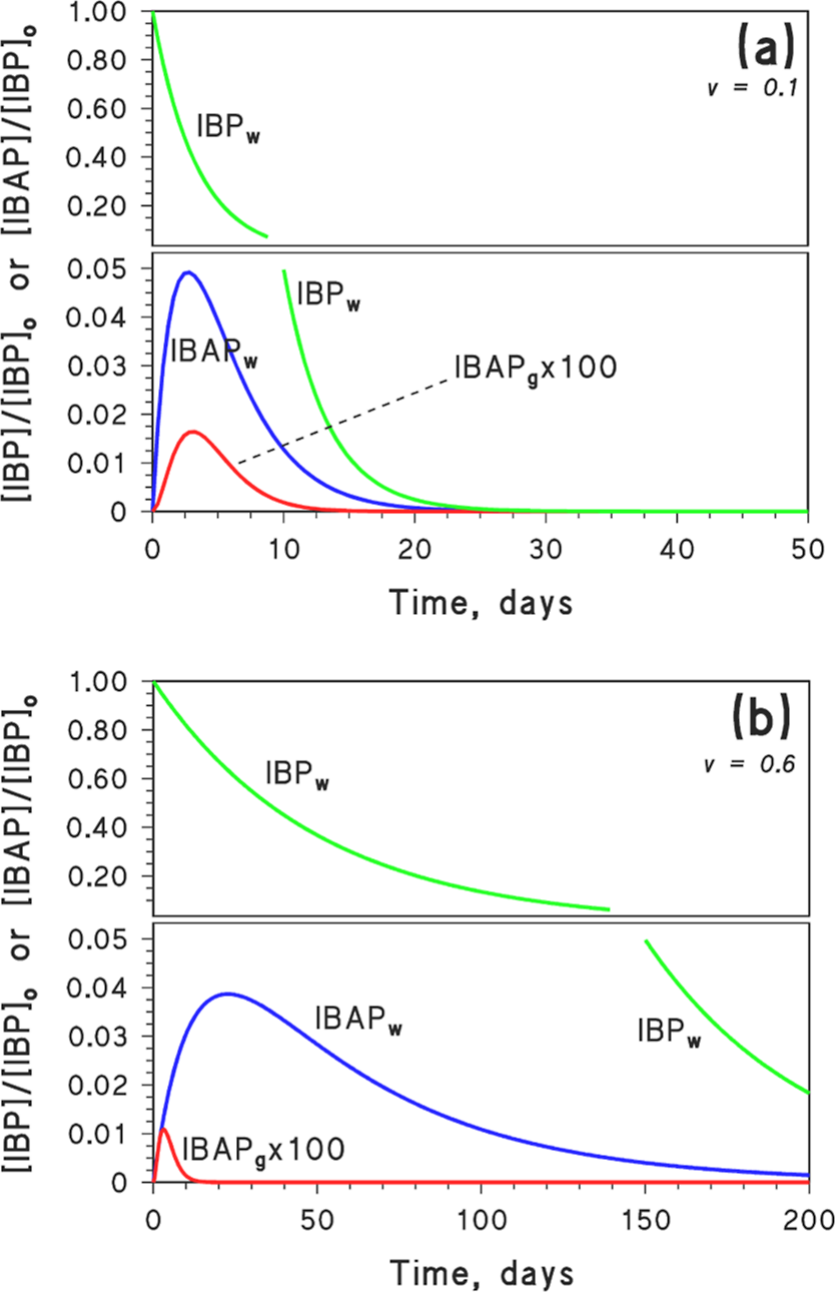
Time trends of IBP_w_, IBAP_w_, and IBAP_g_ based on eqs 6–8 with the following parameters: (a) *k*_d_ = 0.3 day^–1^, *k*_f_ = 0.05 day^–1^, *k*_d_^′^ = 0.4 day^–1^, *k*_vol_ = 0.052 day^–1^, and *k*_g_ = 0.62 day^–1^; (b) *k*_d_ = 0.02 day^–1^, *k*_f_ = 0.005 day^–1^, *k*_d_^′^ = 0.03 day^–1^, *k*_vol_ = 0.052 day^–1^, and *k*_g_ = 0.62 day^–1^. Note the break in the *Y*-axis and the fact that the concentration of IBAP_g_ was multiplied by 100.

To transfer these results into the two-phase model depicted in Scheme 1, we assumed the following scenarios for the aqueous-phase transformation and inter-conversion of IBP and IBAP: (i) “fast” kinetics, with *k*_d_ = 0.3 day^–1^, *k*_f_ = 0.05 day^–1^, and *k*_d_^′^ = 0.4 day^–1^ (*d* = 1 m, DOC = 1 mg_C_ L^–1^, high NO_*x*_^–^); (ii) “slow” kinetics, with *k*_d_ = 0.02 day^–1^, *k*_f_ = 0.005 day^–1^, and *k*_d_^′^ = 0.03 day^–1^ (*d* = 10 m, DOC = 10 mg_C_ L^–1^, low NO_*x*_^–^). In all the cases, *k*_vol_ = 0.052 day^–1^ and *k*_g_ = 0.47 day^–1^. The corresponding time trends of IBP_w_, IBAP_w_, and IBAP_g_ (eqs 6–8) are shown in Figure 5 (5a: fast kinetics; 5b: slow kinetics). The plots suggest that IBAP_w_ is less stable than IBP_w_, and it would thus not occur in the aqueous phase after IBP disappearance (this finding is different from the Henry’s law equilibrium approach: compare Figure 5 with S7 in Supporting Information). Therefore, IBP degradation would also entail disappearance of its toxic transformation intermediate by both volatilization and photodegradation.

The fraction *v* of IBAP that undergoes volatilization to the gas phase is inversely proportional to the transformation kinetics of IBAP in aqueous solution: indeed, the longer the IBAP_w_ persists, the more chances it has to volatilize. In particular, *v* would range from 10% in the “fast kinetics” scenario (Figure 5a) to 60% in the “slow kinetics” one (Figure 5b). In the latter case, more than half of IBAP would escape to the gas phase, where it would be degraded by ^•^OH_(g)_. Because the reaction IBAP + ^•^OH_(g)_ is fast, the gas-phase levels of IBAP would always be extremely low, irrespective of the *v* value.

The fraction *F*_*j*_ of IBAP that is degraded by a given process *j* (direct photolysis, ^•^OH, or ^3^CDOM* in water, or gas-phase ^•^OH after volatilization) can be expressed as follows
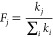
11where *k*_*i*_ = *k*_^•^OH_, *k*_^3^CDOM*_, *k*_d.p._, or *k*_vol_ represents the first-order rate constants of IBAP removal by the processes that were taken into account here. Overall, the photodegradation routes of IBAP in the two scenarios would be the following (note that the two scenarios assume different DOC and NO_*x*_^–^ values, which modify the photodegradation pathways of IBAP in water and, therefore, the values of *k*_^•^OH_, *k*_^3^CDOM*_, and *k*_d.p._):

(i) Fast aqueous-phase kinetics (shallow water with high NO_*x*_^–^ and low DOC): 45% IBAP would be degraded by direct photolysis in water, 40% by reaction with ^•^OH_(w)_, 5% by ^3^CDOM_(w)_^*^, and 10% by ^•^OH_(g)_.
(ii) Slow aqueous-phase kinetics (deep water with low NO_*x*_^–^ and high DOC): 60% IBAP would be degraded by reaction with ^•^OH_(g)_, 30% by ^3^CDOM_(w)_^*^, and 5% each by ^•^OH_(w)_ and direct photolysis in water.

These data suggest that IBAP volatilization to the gas phase, and subsequent degradation by ^•^OH_(g)_, cannot be ignored, especially in the case of deep water bodies with high DOC. Note that *d* = 10 m could well be the epilimnion depth of a deep lake during the summer season.^57^ In such a scenario, IBAP volatilization (followed by gas-phase degradation) is expected to contribute much to IBAP removal from water. Still, gas-phase IBAP degradation is so fast compared to volatilization that it would be very unlikely to detect IBAP in the atmosphere.

## Environmental Significance

IBAP is formed in sunlit surface waters upon photodegradation of IBP by ^3^CDOM* (31% IBAP yield), direct photolysis (25% yield) and, with lower importance, ^•^OH reaction (2.3% yield). Once photoproduced, IBAP can undergo photodegradation in water, as well as partition to the gas phase. Interestingly, IBAP would quickly react with gas-phase ^•^OH, showing half-life times of 1.5–2 days that are faster/much faster than those in sunlit surface waters. The latter amount to 2–30 days depending on conditions such as depth and water chemistry (especially the DOC content). The combination of relatively slow volatilization with quite fast transformation kinetics could hamper detection of IBAP in the gas phase. Actually, Figure 5b suggests that while IBP and IBAP could persist in water for several months, IBAP would disappear from the atmosphere after around 10 days. Although detection of gas-phase IBAP might be difficult, it is still possible to assess the fraction of volatilized IBAP by means of the parameter *v* = *k*_vol_/(*k*_vol_ + *k*′_d_).

**Figure 6:**
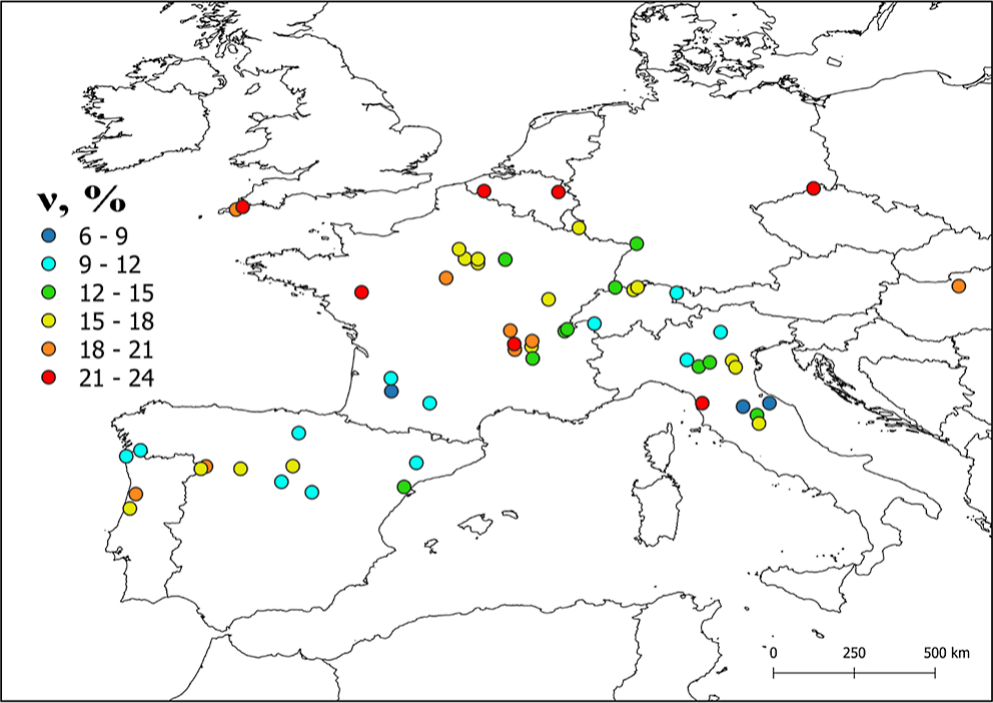
European map of the parameter *v* (%) for IBAP, as obtained from the adopted photochemical model (assuming 2 m water depth).

Figure 6 shows the calculated values of *v* for some European rivers, located in the latitude belt between 40 and 50°N. Details about the modeling procedure are reported in Text S2. It was considered a reasonable 2 m deep water column, while DOC ranged from <1 to ∼10 mg_C_ L^–1^ (nitrate and nitrite were not included in the model, because such data are lacking, which might slightly underestimate photoreaction kinetics).^58^ It is suggested that IBAP volatilization from surface-water environments to the gas phase is potentially quite significant as it may approach up to 25% for the considered scenarios. Higher values of *v* were found in rivers with a relatively high content of (C)DOM (DOC ≥ 7 mg_C_ L^–1^), where IBAP direct photolysis would be inhibited by CDOM (which absorbs sunlight) and only partially offset by the prevailing ^3^CDOM* degradation. For lower DOC values (<5 mg_C_ L^–1^), faster IBAP photodegradation would decrease the relative role of volatilization. Interestingly, direct photolysis and ^3^CDOM* reaction would be comparable for 5 mg_C_ L^–1^ < DOC < 7 mg_C_ L^–1^ and, in these conditions, reaction with ^•^OH_(w)_ would account for ∼10% IBAP degradation. Variation of the water depth, in the range of 1–3 m, would affect the IBAP fraction undergoing partitioning to the gas phase (see Figure S8 in Supporting Information).

The photochemical transformation of ionizable IBP into semivolatile IBAP might not be a general finding for all contaminants. In several instances, the phototransformation products of pollutants are hydroxylated compounds and/or molecules bearing a carboxylic function that makes them less volatile than their parent pollutant. However, there are also several cases in which a pollutant yields more volatile phototransformation products: some examples are the transformation of clofibric acid into 4-chlorophenol by ^•^OH and ^3^CDOM*; of diclofenac into 2,6-dichloroaniline by ^3^CDOM*,^59^ and of gemfibrozil into 2,5-dimethylphenol by direct photolysis.^60^

## Supporting Information Available

Gas-phase reactivity between IBAP and ^•^OH, as well as the modeling of IBAP photochemistry in water; description of an alternative approach based on gas–water partitioning equilibrium (Henry’s law); and detailed procedure used to map IBAP volatilization in European rivers (PDF)
