# Supplementary material for: Evaluation of the Environmental Fate of a Semivolatile Transformation Product of Ibuprofen Based on a Simple Two-Media Fate Model
Source: Environ Sci Technol. 2022 Oct 14;56(22):15650–60. doi: 10.1021/acs.est.2c04867 (PMC9670848; doi:10.1021/acs.est.2c04867)
Supplement: Supplementary file 1 — es2c04867_si_001.pdf [file es2c04867_si_001.pdf]

Supporting Information

Evaluation of the environmental fate of a semivolatile transformation product of ibuprofen, based on a simple two-media fate model

Cecilia Arsene,[1,2,3] Iustinian G. Bejan,[1,2,3] Claudiu Roman,[1,2,3] Romeo I. Olariu,[1,2,3]* Marco Minella,[4] Monica Passananti,[4,5] Luca Carena,[4] Davide Vione[4]*

1 - Department of Chemistry, Faculty of Chemistry, "Alexandru Ioan Cuza" University of Iasi, 11 Carol I, 700506 Iasi, Romania

2 - Integrated Centre of Environmental Science Studies in the North Eastern Region (CERNESIM), "Alexandru Ioan Cuza" University of Iasi, 11 Carol I, 700506 Iasi, Romania

3 - Integrated Centre of Environmental Science Studies in the North Eastern Region (RECENT AIR), "Alexandru Ioan Cuza" University of Iasi, 11 Carol I, 700506 Iasi, Romania

4 - Dipartimento di Chimica, Università degli Studi di Torino, Via Pietro Giuria 5, 10125 Torino, Italy

5 - Institute for Atmospheric and Earth System Research/Physics, Faculty of Science, University of Helsinki, FI-00014, Finland

* Address correspondence to either author. *oromeo@uaic.ro* (RIO); *davide.vione@unito.it* (DV)

Table of contents (12 pages and 8 figures)

List of abbreviations	S2
Figure S1, Figure S2	S3
Figure S3, Figure S4	S4
Figure S5	S5
Figure S6	S6
Text S1	S7
Figure S7	S9
Text S2	S10
Figure S8	S11
References	S12

List of abbreviations

DOC: Dissolved Organic Carbon

IBAP: 4-Isobutylacetophenone

IBP: Ibuprofen

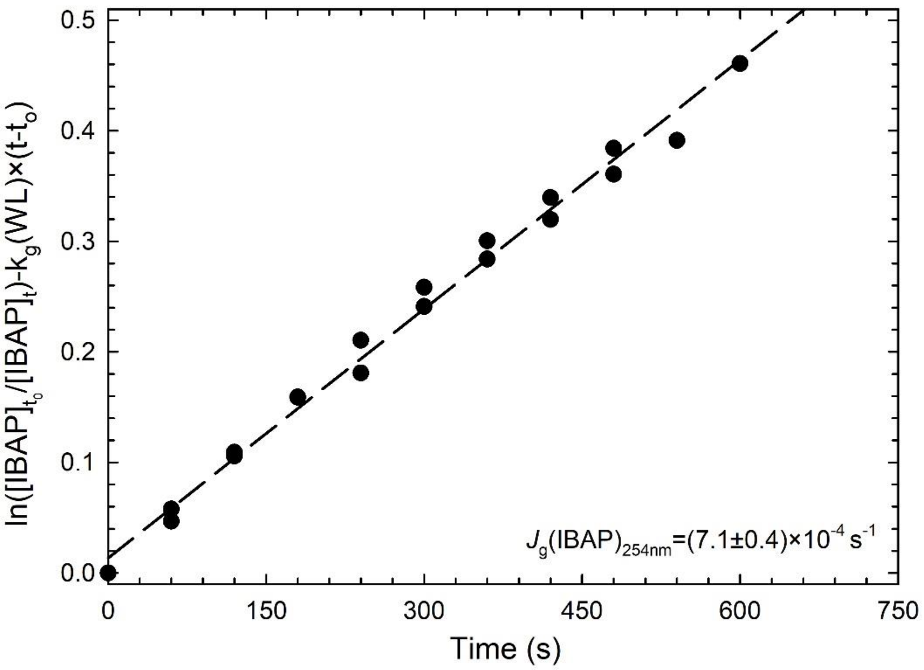

Figure S1. Plot of 254 nm photolysis of IBAP in the ESC-Q-UAIC reactor, corrected for wall loss.

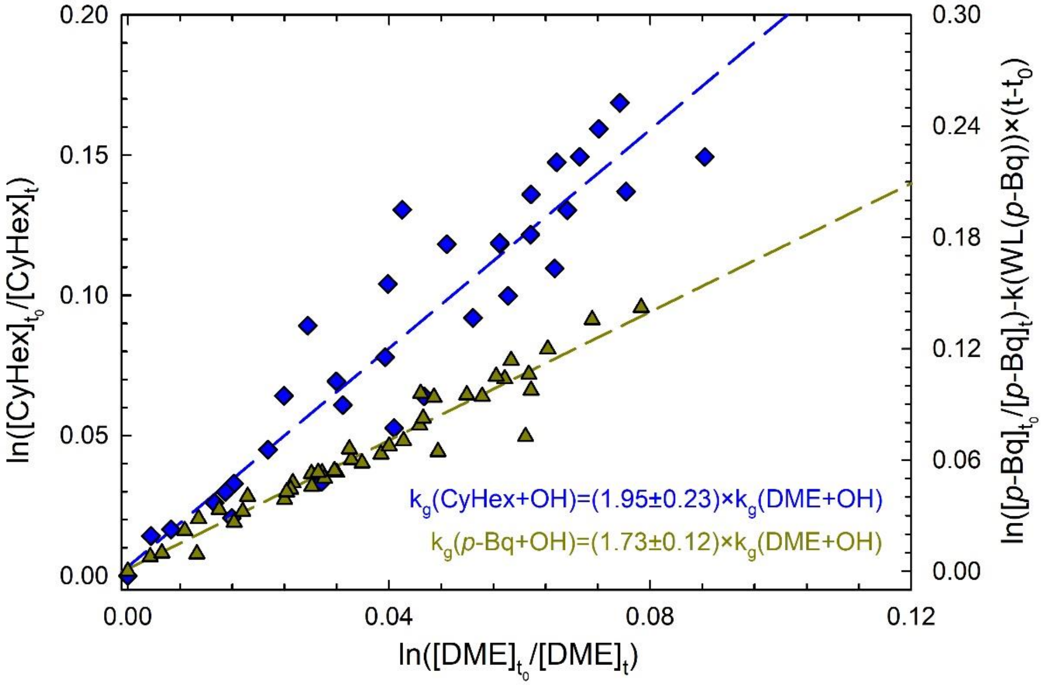

Figure S2: Relative kinetic control plot, corresponding to the gas-phase reaction of •OH radicals with (◆) cyclohexane and (▲) *p*-benzoquinone, versus dimethyl ether in the presence of NOx.

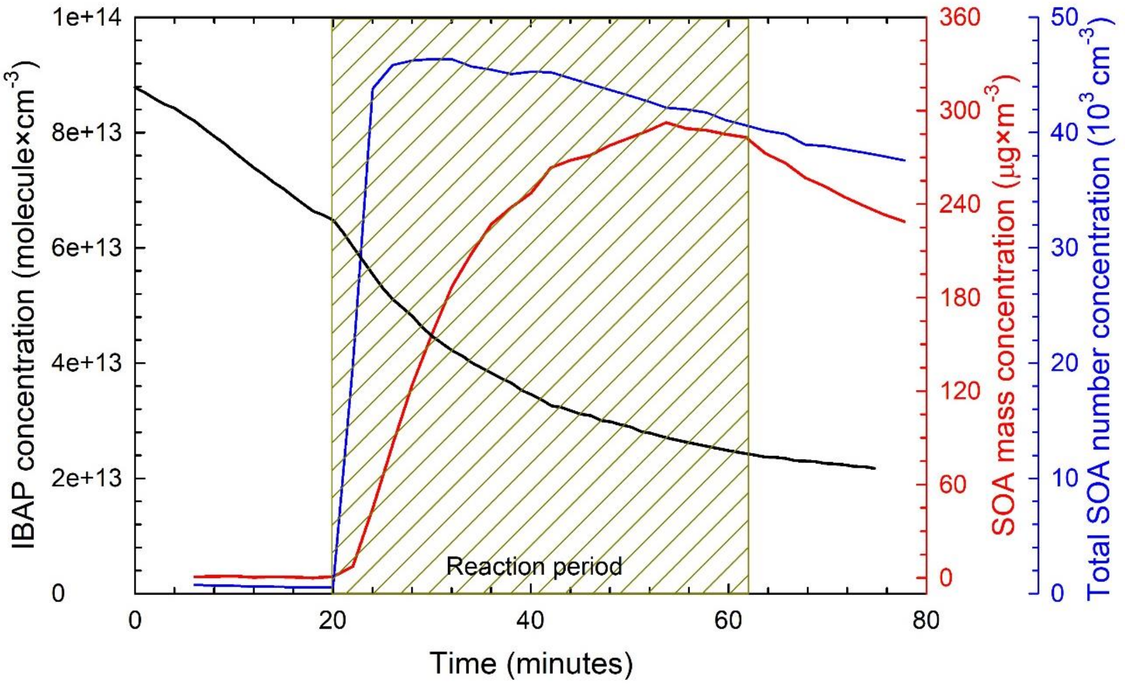

Figure S3: Time profile of IBAP with OH/NOx, and the evolution of the total number and mass concentration of Secondary Organic Aerosol (SOA), in the ESC-Q-UAIC chamber.

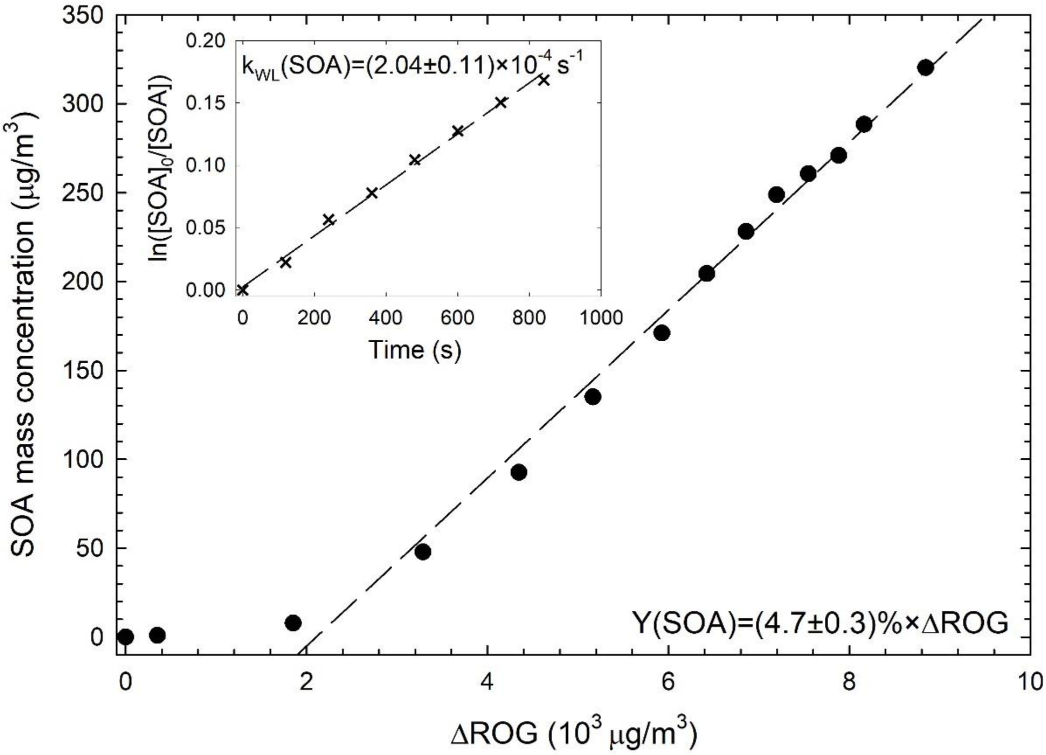

Figure S4: Distribution of the mass concentration of secondary organic aerosols (SOAs) formed in the ESC-Q-UAIC chamber, during the •OH radicals-initiated oxidation of IBAP in the presence of NOx, as a function of IBAP consumption (ΔROG – mass concentration of the reactive organic (IBAP) consumed during the photooxidation process). The intercept with the x axis indicates that SOAs formation occurs only when a critical amount of the reactive organic is consumed (ΔROG), and transformed into initial aerosol precursor materials occurring in the gas phase. This issue suggests that the detected particles are of secondary nature.

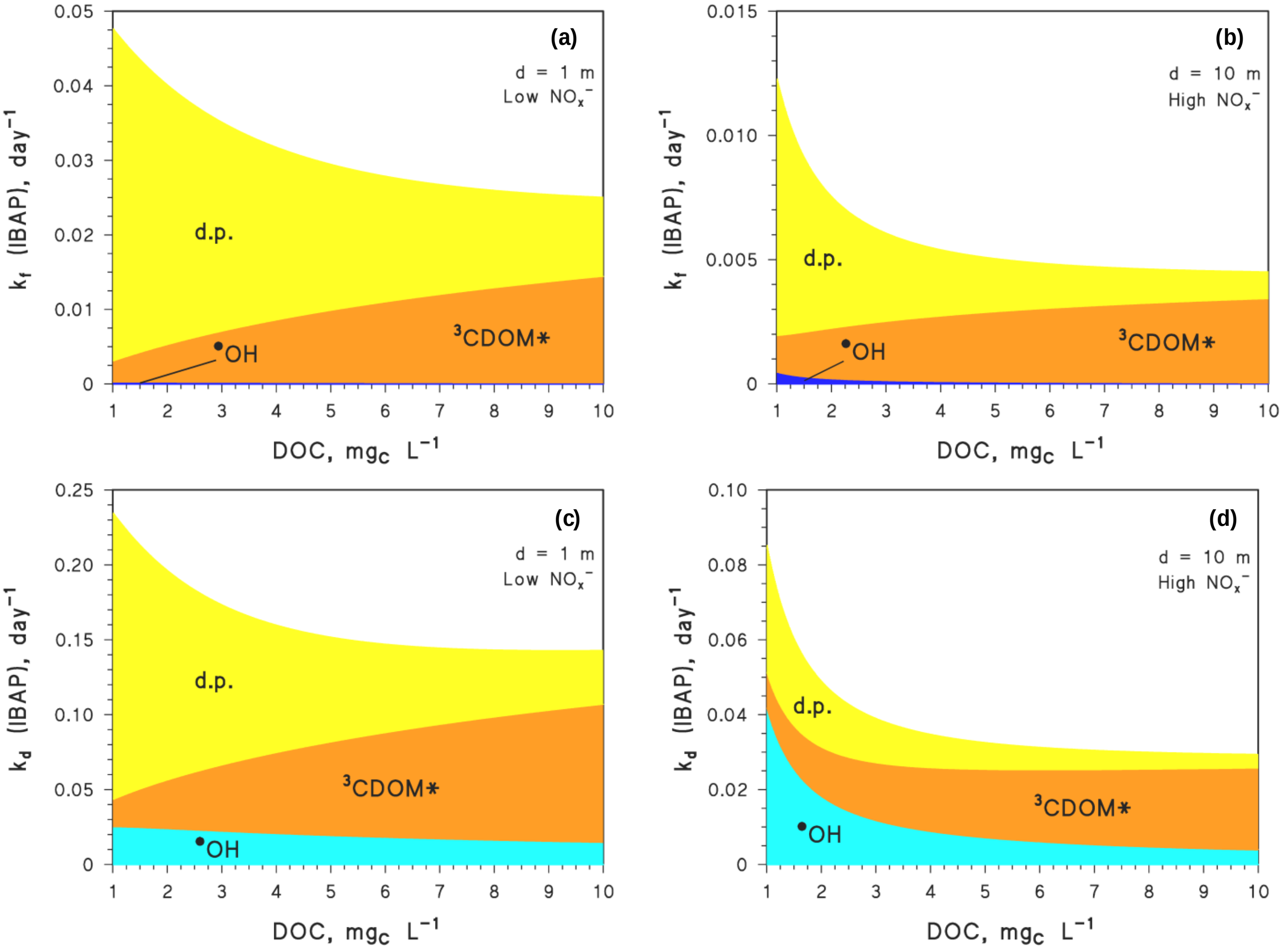

Figure S5. (a,b) Modeled formation rate constants of IBAP from IBP, as a function of water DOC. Other conditions: 10^{-3} M HCO_3^-; 10^{-5} M CO_3^{2-}; water depth *d* ***(a)*** 1m, ***(b)*** 10 m; inorganic nitrogen ***(a)*** $[NO_3^-] = 10^{-6}$ M & $[NO_2^-] = 10^{-8}$ M (low NO_x^-), ***(b)*** $[NO_3^-] = 10^{-4}$ M & $[NO_2^-] = 10^{-6}$ M (high NO_x^-). **(c,d)** Modelled photodegradation rate constants of IBAP, as a function of water DOC. Other conditions: 10^{-3} M HCO_3^-; 10^{-5} M CO_3^{2-}; water depth *d* ***(c)*** 1m, ***(d)*** 10 m; inorganic nitrogen ***(c)*** $[NO_3^-] = 10^{-6}$ M & $[NO_2^-] = 10^{-8}$ M (low NO_x^-), ***(d)*** $[NO_3^-] = 10^{-4}$ M & $[NO_2^-] = 10^{-6}$ M (high NO_x^-). In all the cases, photochemical modelling was carried out with the APEX software.[S1] Average sunlight irradiance & day unit correspond to fair-weather 15 July at 45°N latitude. The color code highlights the different photochemical reaction pathways (d.p. = direct photolysis).

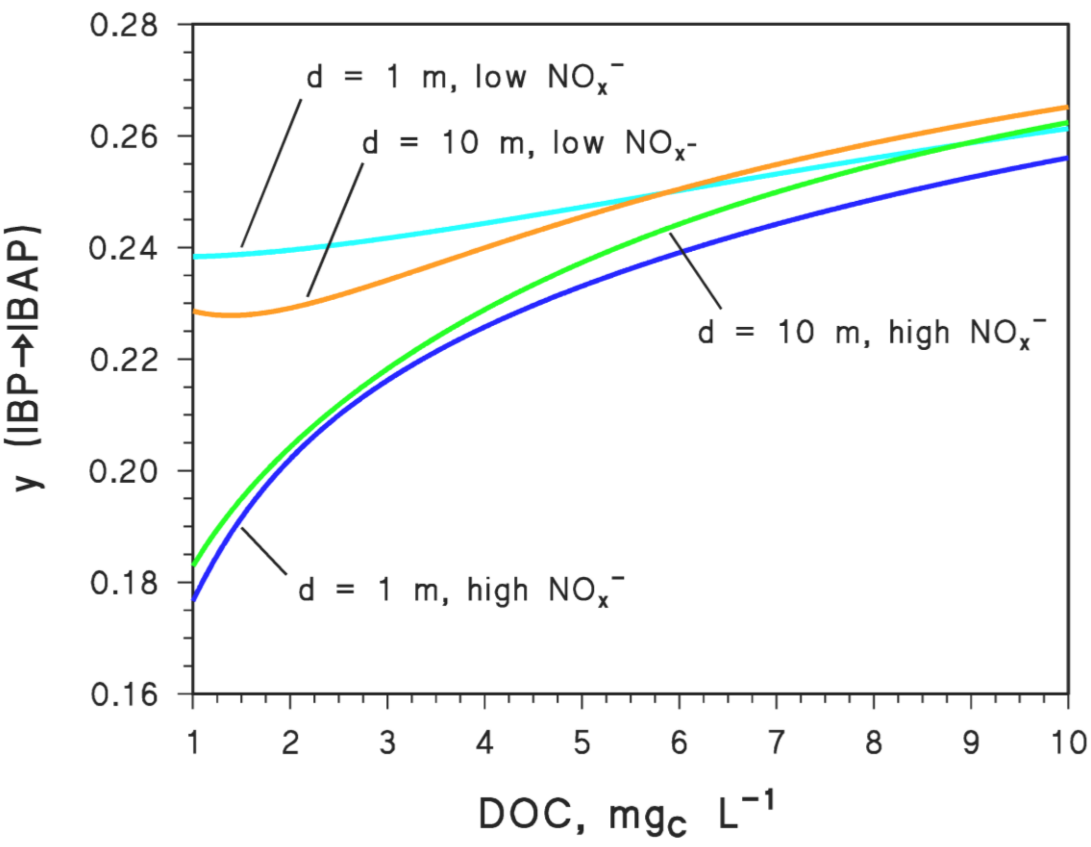

Figure S6. Modeled formation yields of IBAP from IBP, as a function of water DOC. Water conditions: 10^{-3} M HCO_3^-; 10^{-5} M CO_3^{2-}; water depth *d* = 1 m or 10 m. High NO_x^-: $[NO_3^-] = 10^{-4}$ M & $[NO_2^-] = 10^{-6}$ M; low NO_x^-: $[NO_3^-] = 10^{-6}$ M & $[NO_2^-] = 10^{-8}$ M. Sunlight irradiation conditions correspond to fair-weather 15 July at 45°N latitude.

Text S1: An equilibrium approach for IBAP volatilization, based on the Henry's law

This approach is based on the partitioning equilibrium of IBAP, under the hypothesis that gas-phase partitioning is much faster than IBAP transformation in either the gas or the aqueous phase:

$$IBAP_w \leftrightarrows IBAP_g \qquad (S1)$$

EpiSuite™ yielded a value of K_H = 3.4×10^{-5} atm m^3 mol^{-1} for the Henry's law constant of IBAP, which would be the ratio between IBAP's partial pressure in the gas phase and its concentration in aqueous solution: $K_H = p_g / c_w$ (note that, in this case, the measure unit of c_w is [mol m^{-3}]). Here, it is more convenient to express the Henry's law constant in unitless form, as $H = c_g / c_w$, where both IBAP concentrations are expressed in [mol L^{-1}]. From the gas law, one has that $p_g V_g = n_g$ R T and that, as a consequence, $c_g = n_g / V_g = p_g / (RT)$. Therefore, $K_H = RT/1000 \; c_g / c_w = (RT/1000)H$ = 2.45×10^{-2} H, where 1000 is the conversion factor between [mol m^{-3}] and [mol L^{-1}]. As a consequence, in the case of IBAP, one gets H = 1.4×10^{-3}.

If the partitioning equilibrium is much faster than IBAP transformation, one has to consider a lumped disappearance kinetics of the compound in both phases (water and air), taken together. In this case, the decay rate constant of IBAP (k) is the average of the gas- and aqueous-phase decay constants (k_g and k_w, respectively), weighted for the number of IBAP moles occurring in each phase (n_g and n_w, respectively):

$$k = k_g \, n_g / n_{tot} + k_w \, n_w / n_{tot} = k_g \frac{c_g V_g}{c_g V_g + c_w V_w} + k_w \frac{c_w V_w}{c_g V_g + c_w V_w} \qquad (S2)$$

where $n_{tot} = n_g + n_w$, V_g is the volume of the gas phase, and V_w that of the aqueous solution. The time evolution of IBP and IBAP can be expressed as follows (k' is IBP's pseudo-first order decay constant in water, while c' and c_o' represent its molar concentrations in water at time t and time 0, respectively; in the case of IBAP, k_f is the formation rate constant from IBP in water, while c_T is IBAP's concentration value in water and air taken together; k is given by equation S2):

$$\text{IBP:} \quad c' = c'_o \, e^{-k't} \qquad (S3)$$

$$\text{IBAP:} \quad c_T = \frac{k_f \, c'_o}{k - k'} (e^{-k't} - e^{-kt}) \qquad (S4)$$

Note that IBP occurs in water, while the time trend of IBAP considers its lumped occurrence in both phases. On this basis, $c_T = n_{tot} (V_g + V_w)^{-1}$; furthermore, by considering that $n_{tot} = c_g V_g + c_w V_w$, assuming $Q = V_g/V_w$, and remembering that $H = c_g / c_w$, one gets the following for IBAP:

$$c_w = \frac{Q+1}{1+HQ} \frac{k_f\, c_o'}{k-k'} (e^{-k't} - e^{-kt}) \tag{S5}$$

$$c_g = H\, c_w = \frac{H(Q+1)}{1+HQ} \frac{k_f\, c_o'}{k-k'} (e^{-k't} - e^{-kt}) \tag{S6}$$

$$k = k_g \frac{HQ}{1+HQ} + k_w \frac{1}{1+HQ} \tag{S7}$$

From these equations, it is clear that IBAP concentrations in the gas phase and in aqueous solution run parallel ($c_g = H\, c_w$). This is very different from the time trends reported in **Figure 5** of the main manuscript. To make things clearer, assume two different scenarios (fast and slow kinetics in aqueous solution):

1) Fast kinetics: $k' = 0.3$ day^{-1}, $k_f = 0.05$ day^{-1}, $k_w = 0.4$ day^{-1}, and $k_g = 0.62$ day^{-1};
2) Slow kinetics: $k' = 0.02$ day^{-1}, $k_f = 0.005$ day^{-1}, $k_w = 0.03$ day^{-1}, and $k_g = 0.62$ day^{-1}.

It is $H = 1.4\times10^{-3}$ as derived before, and it is here assumed $Q = 10$. One gets $k = 0.4$ day^{-1} in the 'fast kinetics' scenario, and $k = 0.04$ in the 'slow kinetics' one. In both cases, the majority of IBAP would be degraded in aqueous solution. Based on these data, the time trends of IBP, $IBAP_w$, and $IBAP_g$ are shown in **Figure S7**, in the 'fast kinetics' scenario (**S7a**) and in the 'slow kinetics' one (**S7b**). The trends are quite different from those reported in **Figure 5**; the main reasons for the difference are as follows:

(i) In the equilibrium approach, degradation in water is the main driving force for IBAP transformation. Actually, both water degradation and volatilization contribute to IBAP removal from water. Therefore, the equilibrium approach underestimates the decrease of $IBAP_w$ over time.

(ii) The equilibrium approach assumes that gas-water partitioning of IBAP is much faster than IBAP transformation in water. Because, in contrast, the two processes have comparable kinetics, the equilibrium approach based on the Henry's law constant does not provide an accurate representation of the behavior of IBAP.

(iii) In the equilibrium approach, IBAP time trends are predicted to be proportional in both the gas phase and in water ($c_g = H\, c_w$). As shown in **Figure 5** of the main manuscript, this is not always an accurate description of the system.

(iv) According to the equilibrium approach with $Q = 10$, only 2-22% of IBAP transformation would occur in the gas phase. The actual percentage is 10-60%. For the equilibrium approach to produce the same results, one has to assume $Q = 700$ (the choice of Q is clearly

quite arbitrary). However, in this case one would overestimate the degradation rate constant of IBAP in the water phase (by up to one order of magnitude, in the case of slow kinetics).

For the reasons mentioned above, a partitioning equilibrium approach based on the Henry's law does not provide an accurate description of the behavior of IBP/IBAP in a coupled water/atmosphere system.

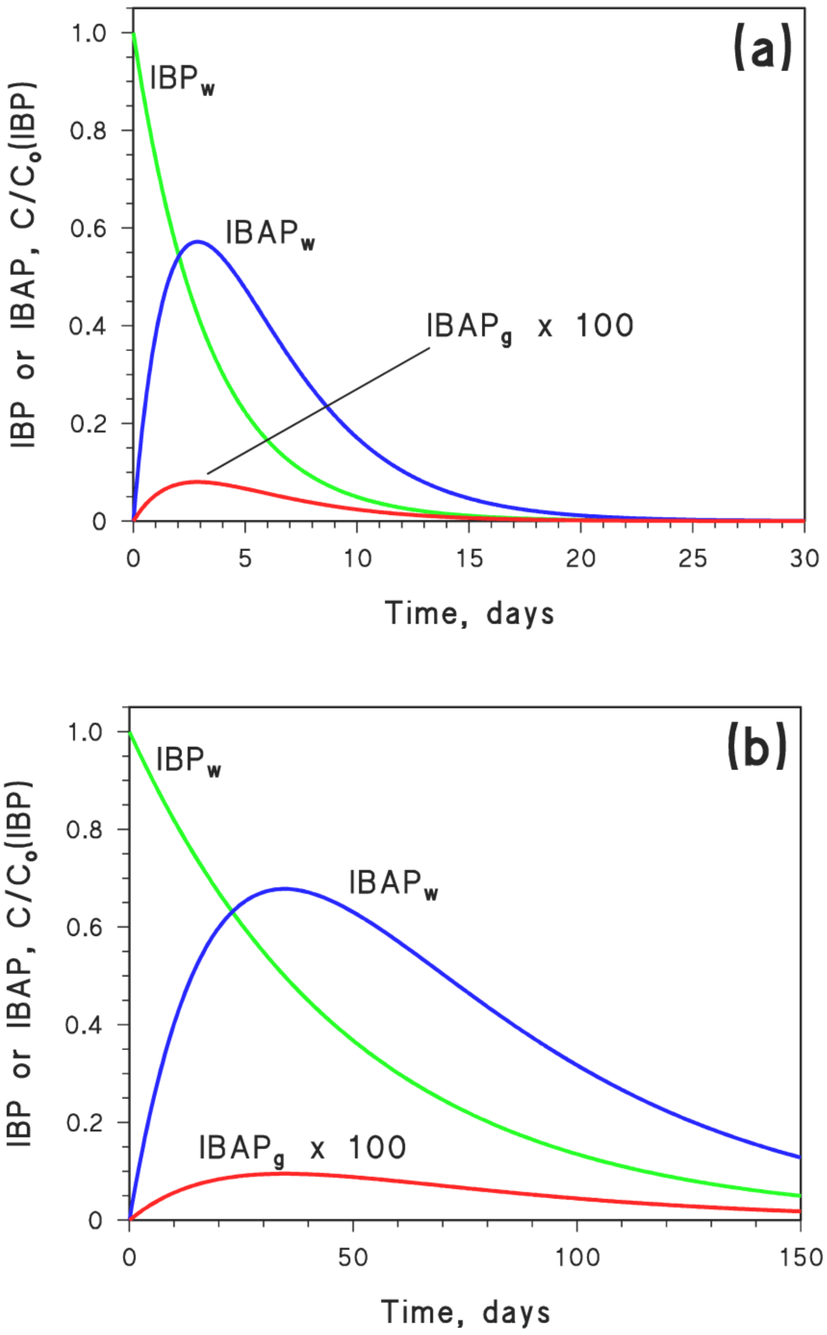

Figure S7. Time trends of IBP_w, $IBAP_w$, and $IBAP_g$, based on equations (S3, S5, S6), with the following parameters: **(a)** $k' = 0.3$ day^{-1}, $k_f = 0.05$ day^{-1}, and $k = 0.4$ day^{-1}; **(b)** $k' = 0.02$ day^{-1}, $k_f = 0.005$ day^{-1}, and $k = 0.04$ day^{-1}. Note that the concentration of $IBAP_g$ was multiplied by 100.

Text S2. Mapping of the parameter ν for IBAP volatilization.

As described in the main manuscript, IBAP can undergo both photodegradation in, and volatilization from, surface-water environments. The fraction of IBAP that volatilizes is defined as $\nu = k_{vol}\,(k_{vol} + k'_d)^{-1}$, where k_{vol} is the volatilization rate constant of IBAP from an aqueous environment to the gas phase, and k'_d is the overall photodegradation rate constant of IBAP in water.

We calculated the value of ν for some European rivers, and mapped it over the region included between 40 and 50°N during the month of June, in the decade 1990 - 2000. The values are averaged over that period. Photochemical computation of k'_d was mainly based on the values of the DOC (Dissolved Organic Carbon, [$mg_C\ L^{-1}$]) of the considered rivers.[S2] This means that the photolysis rate constant k'_d was assessed, without considering the photochemistry of nitrate and nitrite. For a complete description of data sources and treatment, the reader is referred to Ref. **S3**. Briefly, the overall rate coefficient of IBAP photodegradation is mainly accounted for by reactions with $^{\bullet}OH$ radicals and $^3CDOM^*$, as well as by direct photolysis. Therefore, for a given river, one has that $k'_d = k_{IBAP,{}^3CDOM^*}[{}^3CDOM^*] + k_{IBAP,{}^{\bullet}OH}[{}^{\bullet}OH] + \Phi \times P'_a(IBAP)$, where the values of $k_{IBAP,{}^3CDOM^*}$, $k_{IBAP,{}^{\bullet}OH}$, and Φ are reported in **Table 1** of the main manuscript. $P'_a(IBAP)$ is the rate coefficient of solar-light absorption by IBAP in the considered river, and it was assessed as follows:

$P'_a(IBAP) = \Sigma_\lambda\, p°(\lambda)\, \varepsilon_{IBAP}(\lambda)\, [1 - 10^{-A_1(\lambda)\, l\, DOC}](A_1(\lambda)\, DOC)^{-1}\, \Delta\lambda$.

In particular, $p°(\lambda)$ is the spectral solar photon flux density (Ein $L^{-1}\ s^{-1}\ nm^{-1}$) reaching the water body in mid-June, $\varepsilon_{IBAP}(\lambda)$ is the molar absorption coefficient of IBAP (L $mol^{-1}\ cm^{-1}$), $A_1(\lambda) = 0.45\ \exp(-0.015\ \lambda)$ [S1] is the specific absorbance of CDOM along an optical path length of 1 cm (units of L $mg_C^{-1}\ cm^{-1}$), and l (cm) is the river optical path length (which is a function of the water depth [S1]). [$^3CDOM^*$] and [$^{\bullet}OH$] were calculated with APEX,[S1] from the DOC values of the considered rivers. With the values of k'_d thus calculated (units of s^{-1}), as well as $k_{vol} = 0.052\ day^{-1} = 6.02\cdot10^{-7}\ s^{-1}$, the parameter ν was computed and mapped over the European region, by means of the QGIS software, version 3.2.2 'Bonn' (QGIS Developmental Team, 2020. QGIS Geographic Information System. Open Source Geospatial Foundation Project; 2018). **Figure S8** shows the calculated values of ν for water depths of 1 and 3 m. The intermediate case (2 m) is shown in the main manuscript.

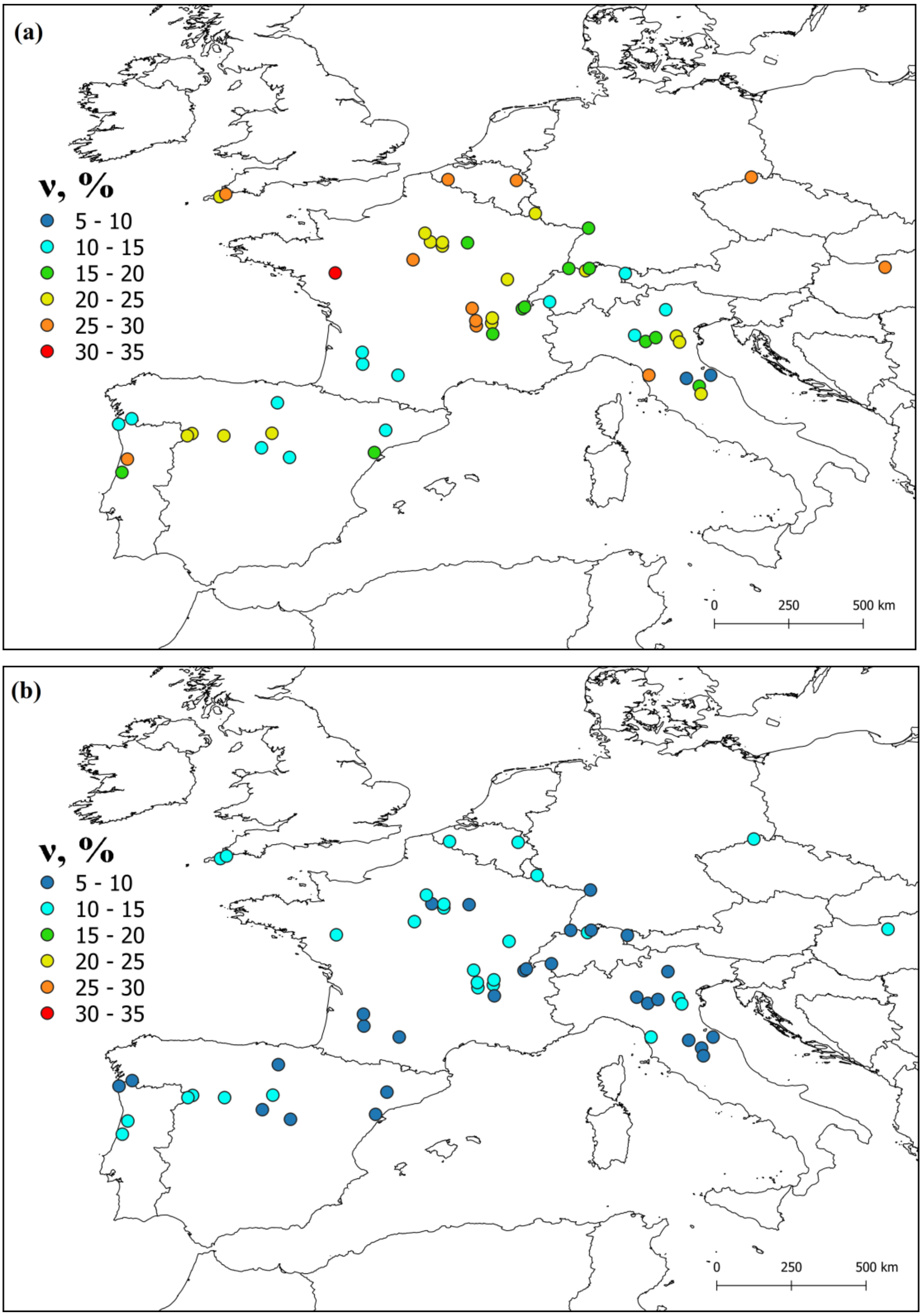

Figure S8. European maps of the parameter ν (%) for IBAP, as obtained from the adopted photochemical model, for water depth = 1 m **(a)** and 3 m **(b)**.

References

S1) Bodrato, M.; Vione, D. APEX (Aqueous Photochemistry of Environmentally occurring Xenobiotics): A free software tool to predict the kinetics of photochemical processes in surface waters. *Environ. Sci.-Proc. Imp.* **2014**, *16*, 732-740.

S2) GEMSTAT, http://www.GEMStat.org.

S3) Carena, L.; Vione, D. Mapping the photochemistry of European mid-Latitudes rivers: An assessment of their ability to photodegrade contaminants. *Molecules* **2020**, 25.
